# Over-the-Air Performance Evaluation of an Open-Source Private 5G SA Network for B5G Experimentation

**DOI:** 10.3390/s26175355

**Published:** 2026-08-24

**Authors:** Valentin Popa, Adrian I. Petrariu, Alexandru A. Maftei, Partemie M. Mutescu, Alexandru Lavric, Razvan Marius Mihai, Cristian Pațachia Sultanoiu

**Affiliations:** 1 Computers, Electronics and Automation Department, Stefan cel Mare University of Suceava, 720229 Suceava, Romania; valentin@eed.usv.ro (V.P.); apetrariu@usm.ro (A.I.P.); alexandru.maftei@usm.ro (A.A.M.); marian.mutescu@usm.ro (P.M.M.); 2 MANSiD Research Center, Stefan cel Mare University of Suceava, 720229 Suceava, Romania; 3 Faculty of Electronics, Telecommunications and Information Technology, National University of Science and Technology Politehnica Bucharest, 061071 Bucharest, Romania; razvan.mihai@orange.com; 4 Orange Romania, 010144 Bucharest, Romania; cristian.patachia@orange.com; 5 Faculty of Electronics, Telecommunications and Information Technology, Technical University of Iasi Gheorghe Asachi, 700050 Iasi, Romania

**Keywords:** open RAN, 5G standalone, Open5GS, srsRAN, software-defined radio, beyond-5G

## Abstract

The transition from early non-standalone 5G deployments to 5G Standalone and, more recently, 5G-Advanced has turned mobile networks into flexible, programmable infrastructures capable of supporting private, industrial, and research-oriented deployments for the development of beyond-5G applications and architectures. Evaluating these networks’ capabilities, however, remains challenging because commercial platforms often provide limited access to internal interfaces, radio parameters, and network measurements. This paper presents an open-source private 5G SA testbed for beyond-5G application validations built using Open5GS, srsRAN, Ettus USRP N310 software-defined radio, programmable SIM cards, and commercial 5G customer-premise equipment. The platform is deployed in a semi-anechoic chamber. End-to-end operation is validated through subscriber registration, authentication, PDU session establishment, and external data connectivity. The performance of the implemented 5G network is evaluated using throughput, block error rate, modulation and coding scheme, and gNB trace logs. Unlike previous open-source 5G testbeds that primarily use RF waveguides, individual network components, or a limited set of radio configurations, the proposed platform combines COTS SIM-based UE operation with a controlled over-the-air evaluation of FDD/TDD and multiple antenna configurations and correlates application-level throughput with internal gNB radio metrics. For FDD downlink operation, the average throughput increased by approximately 74% from 1 × 1 to 2 × 2 and by a further 57% from 2 × 2 to 4 × 4, although the additional peak-throughput gain from 2 × 2 to 4 × 4 remained limited. The platform provides a reproducible environment for validating beyond-5G mechanisms, comparing network configurations, and studying the behavior of future open-source 5G SA systems under controlled conditions.

## 1. Introduction

Fifth-generation standalone (5G SA) cellular networks have moved beyond early non-standalone (NSA) deployments using a dedicated 5G core network (CN) instead of relying on Long-Term Evolution (LTE) control-plane infrastructure. Compared to NSA deployments, in a 5G SA system, the Radio Access Network (RAN), the CN, and the user-plane forwarding path can be deployed and configured separately [1,2]. This gives the 5G network the ability to perform network slicing, which allows the implementation of low-latency services, flexible user-plane deployment, and non-public networks (NPNs). These capabilities are important for both private 5G deployments and the evaluation of Beyond 5G (B5G) networks, which are expected to require increased programmability, automation, service awareness, and integration between communication, computing, and application functions [3]. Research centers therefore need experimental platforms that provide direct control over network functions, network configuration, and measurements of internal network parameters to design and develop next-generation B5G networks. Open RAN provides access to network telemetry and programmable control interfaces that can support closed-loop AI/ML-based radio optimization [4].

Commercial 5G systems have reached a high level of maturity since their introduction in 2019. However, many operator-grade solutions remain proprietary, making them difficult to use in research applications. In typical vendor systems, the radio unit, baseband processing, and management software are closely integrated into a single platform. This limits access to internal network interfaces, making it hard to bring any modifications to the radio parameters or measurement functions. Open RAN and open-source 5G platforms address this problem by separating software from hardware, enabling deployment on commercial off-the-shelf (COTS) hardware, and providing researchers with a more flexible environment for testing and reconfiguration.

Laboratory-scale 5G testbeds are an important part of research on the improvement of current 5G networks with perspectives towards 6G networks. They allow researchers to validate hypotheses, study the internal behavior of the CN software, and produce repeatable experimental results. Building a complete 5G testbed, however, is still difficult as a full 5G network deployment combines the CN, RAN components, radio frontends, subscriber credentials, routing rules, and traffic measurement tools into one working end-to-end system. The task becomes more complex when the goal is to test real radio access behavior with COTS User Equipment (UE) and over-the-air (OTA) transmission, rather than only to emulate the network.

Beyond-5G research is not limited to increasing radio throughput. It also involves developing intelligent, application-aware networks. Evaluating these concepts requires an operational end-to-end network in which the behavior of the RAN, CN, user-plane functions, and applications can be observed and modified. In this context, intelligent network-management approaches have also been investigated using privacy-preserving deep learning, transfer learning, neuro-fuzzy methods, and deep reinforcement learning for caching, computing-resource allocation, and energy-aware communication optimization [5,6,7,8]. A configurable 5G SA testbed therefore provides an important transitional platform.

Most 5G laboratory experiments are conducted using radio frequency (RF) waveguides, coaxial cables, splitters, attenuators, and multiplexers. These setups are useful because they provide isolation and repeatability, but they do not fully capture the behavior of a real wireless link. In this paper, we evaluate the deployment of a private 5G SA laboratory platform that uses a point-to-point over-the-air radio link inside a semi-anechoic chamber. The chamber allows OTA testing without interfering with telecom operators, while preserving the practical characteristics of free-space radio signal propagation. Compared with a purely RF waveguide setup, this configuration supports repeatable radio experiments under controlled propagation conditions.

The evaluated architecture combines open-source implementations of both the 5G Core and RAN, a software-defined radio (SRD) frontend, programmable subscriber credentials, COTS UE such as 5G CPE (Customer-Premises Equipment), and IP connectivity to an external network for data services. The testbed is intended to demonstrate end-to-end 5G SA operation but also to provide a configurable environment for B5G application and network development. In the implemented workflow, subscriber credentials are provisioned, the UE registers and authenticates with the network, a PDU session is established, and the UE obtains end-to-end data connectivity. Once connectivity has been established, radio parameters, CN functions, and application behavior can be modified across controlled experimental scenarios.

The current evaluation considers channel configuration, duplexing mode, and antenna configuration. The same architecture can be extended to evaluate B5G-enabling concepts such as closed-loop network automation, AI-assisted radio optimization [4], and enhanced network slicing. The platform therefore acts as a bridge between operational 5G SA systems and the experimental development of B5G networks. Compared to other testbeds that focus on conducted RF connections, individual network components, or a limited number of radio configurations, the proposed setup enables comparative FDD/TDD and 1 × 1, 2 × 2, and 4 × 4 evaluations while evaluating application-level throughput and internal radio metrics such as BLER, MCS, and gNB traces.

The main contributions of this paper are as follows:

- An end-to-end open-source private 5G SA testbed integrating Open5GS, srsRAN, an Ettus USRP N310, programmable SIM cards, and commercial 5G CPE, designed as an extensible tool for B5G networks development and evaluation.
- A repeatable OTA evaluation methodology implemented in a semi-anechoic chamber and covering FDD and TDD operation together with 1 × 1, 2 × 2, and 4 × 4 antenna configurations.
- A performance analysis that combines application-level throughput with BLER, MCS, and gNB trace logs to relate service performance to internal radio and network behavior.

The remainder of the paper is organized as follows. Section 2 introduces the evolution and structure of the 5G networks and Open RAN concept, including Open RAN disaggregation, open-source 5G implementations, SDR-based radio access, and chamber-based OTA testing. Section 3 reviews related work on private 5G, Open RAN, and open-source testbeds. Section 4 presents the testbed design and methodology, including the hardware, software, subscriber configuration, semi-anechoic chamber setup, and measurement scenarios. Section 5 reports and discusses functional validation. Section 6 concludes the paper.

## 2. 5G SA and Open RAN Architecture

### 2.1. 5G Standalone Networks

**Figure 1:**
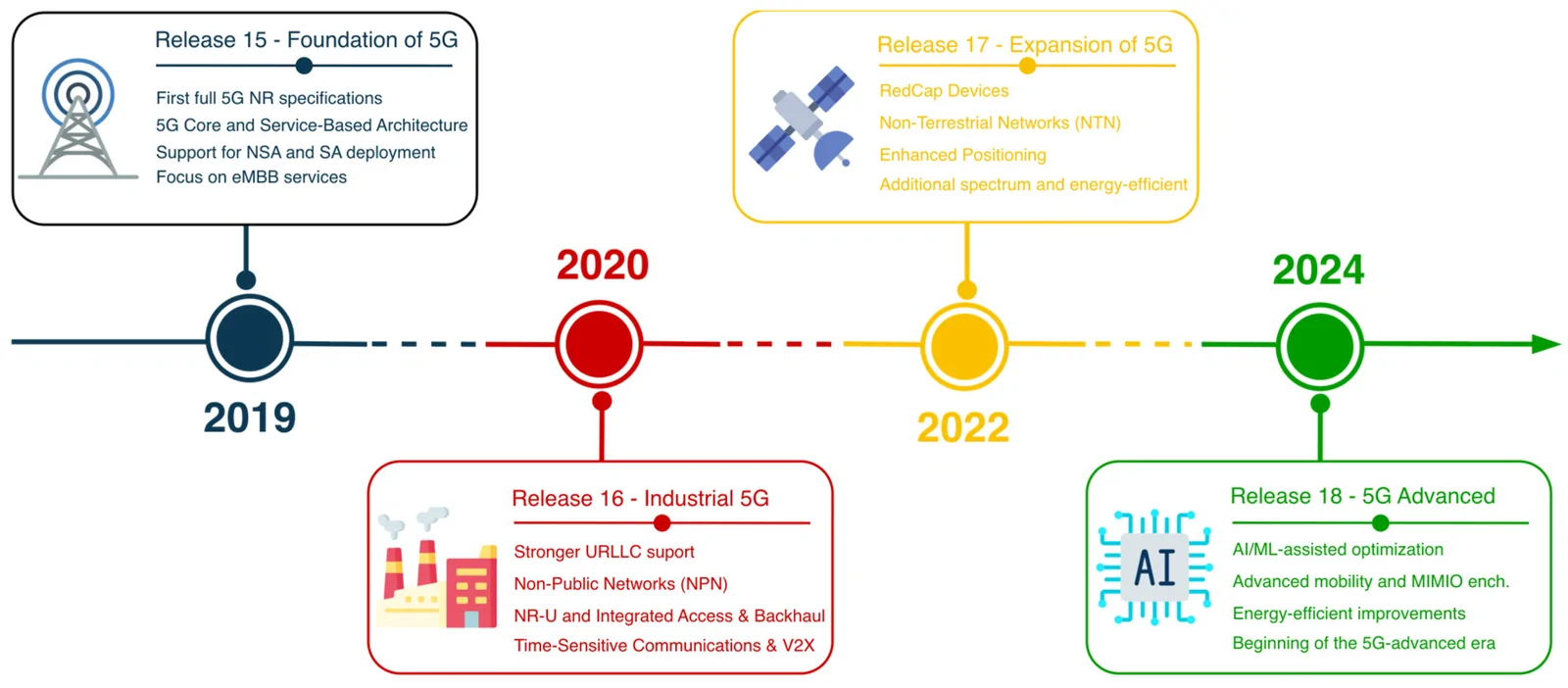
Evolution of 3GPP 5G releases (Releases 15–18), highlighting the major capabilities introduced in each standardized release.

The development of the 5G standard has followed a sequence of 3rd Generation Partnership Project (3GPP) releases, as shown in Figure 1. Release 15 [9] introduced the first complete 5G system, including 5G New Radio (NR), the 5G CN, and SA and NSA deployment modes. Release 15 focused mostly on Enhanced Mobile Broadband (eMMB) services. Following Release 15, the second iteration of the 5G standard, Release 16 [10], added support for industrial use cases through improvements to Ultra-Reliable Low-Latency Communications (URLLCs), Time-Sensitive Communications (TSN), NPN, new radio-unlicensed (NR-U), and Integrated Access and Backhaul (IAB) for 5G network expansion without requiring an optical fiber connection through the use of IAB donor cells, and Vehicle-to-Everything (V2X) services. In 2022, Release 17 [11] added extended support for IoT ecosystems through the introduction of support for Reduced Capability (RedCap) devices, non-terrestrial networks (NTNs), and improvements in UE positioning using network-level measurements. Release 18 [12] marked the beginning of 5G-Advanced, where standardization work covers AI/ML-assisted optimization of network functions, energy efficiency, improvements in high UE mobility scenarios, and advanced automation in RAN and CN [13].

A 5G SA network has three main parts: the UE, the RAN, and the 5G CN. The UE represents the terminal device, such as a smartphone, a 5G modem, an industrial sensor, or CPE. The RAN provides wireless access through the 5G base station, called the gNodeB (gNB). The 5G CN handles network functions such as UE authentication, mobility management, session control, policy enforcement, subscriber management, and connectivity to external data networks. In SA mode, the gNB connects directly to the 5GC and does not rely on a legacy LTE core.

The 5G networks use standardized interfaces between these components. The N1 interface is the logical signaling interface between the UE and the Access and Mobility Management Function (AMF), and it carries Non-Access Stratum (NAS) messages. The N2 interface connects the gNB to the AMF and carries control-plane signaling. The N3 interface connects the gNB to the User Plane Function (UPF) and transports user-plane traffic over the GPRS Tunneling Protocol User Plane (GTP-U). Within the 5G Core, the N4 interface connects the Session Management Function (SMF) to the UPF and controls packet forwarding rules. The N6 interface connects the UPF to an external data network, such as the Internet, an enterprise LAN, or a local application server [14].

The 5G CN is a set of logical network functions, not a single monolithic component. The AMF manages UE registration, access control, mobility, reachability, and NAS signaling. The SMF manages PDU sessions, IP address allocation, and interaction with the UPF. The UPF forwards user-plane packets between the RAN and the data network and acts as the main data-plane function. The Authentication Server Function (AUSF) performs authentication procedures. The Unified Data Management (UDM) and Unified Data Repository (UDR) manage subscriber data and subscription information. The Network Repository Function (NRF) supports service registration and discovery between core functions. The Policy Control Function (PCF) provides policy and QoS control, while the Network Slice Selection Function (NSSF) supports slice selection. This service-based architecture allows the 5G CN to run as separate software functions on general-purpose hardware [14]. This service-based architecture allows the CN to run as separate software functions on general-purpose hardware [14]. This service-based architecture allows the CN to run as separate software functions on general-purpose hardware [14].

### 2.2. Open RAN and gNodeB Disaggregation

RAN is responsible for radio communication between the UE and the 5G CN. In traditional deployments, the base station is usually delivered as a closed vendor-specific system, with hardware and software tightly integrated. This limits interoperability and makes individual components harder to replace or modify. Open RAN addresses this challenge using disaggregation, open interfaces, software-defined processing, and COTS hardware. The main idea behind the Open RAN concept is to reduce vendor lock-in and make RAN deployments easier [15].

In the Open RAN model, gNodeB can be divided into three logical units: the Open Central Unit (O-CU), the Open Distributed Unit (O-DU), and the Open Radio Unit (O-RU). The O-CU hosts higher-layer protocol functions, including Radio Resource Control (RRC), Service Data Adaptation Protocol (SDAP), and Packet Data Convergence Protocol (PDCP). The O-CU handles control signaling, mobility-related procedures, and packet processing. On the other hand, the O-DU performs lower-layer and time-sensitive functions such as Radio Link Control (RLC), Medium Access Control (MAC), and parts of the physical layer (PHY). These functions include scheduling, Hybrid Automatic Repeat reQuest (HARQ) processing, and radio resource allocation. The O-RU contains the lower PHY layer and radio-frequency functions, including digital-to-analog conversion, RF transmission and reception, filtering, amplification, and antenna interfacing.

A significant part of Open RAN is the functional split between the O-DU and O-RU. Among the available split options, Option 7.2x [16] is widely used because it balances fronthaul bandwidth requirements with support for advanced radio functions such as beamforming. With this split, part of the PHY processing remains in software near the O-DU, while the O-RU performs lower PHY and RF operations. This architecture supports multi-vendor interoperability and allows parts of the RAN to be updated, optimized, or replaced without changing the entire base-station system [15]. OpenRAN also adds support for RAN control through the RAN Intelligent Controller (RIC). The Near-Real-Time RIC operates at time scales of approximately 10 ms to 1 s and hosts xApps that optimize radio behavior using near-real-time measurements [17]. The Non-Real-Time RIC is part of the Service Management and Orchestration layer. It operates over longer time scales using rApps and policy-based control. These mechanisms support traffic steering, resource allocation, energy optimization, QoS control, and network slicing. The E2 interface connects RAN nodes to the Near-RT RIC and carries network telemetry measurements, policies, and control commands [15]. It should be noted that the O-RAN architecture and interfaces described in this subsection provide architectural context; the experimental platform evaluated in this work uses an open-source srsRAN-based gNB and does not implement the O-RAN 7.2x, E2, or RIC interfaces.

### 2.3. Open-Source Platforms for Private 5G Deployments

An open-source 5G deployment requires both a 5G CN and an RAN implementation. These two network entities must be kept separate. Open CN platforms implement functions such as AMF, SMF, UPF, AUSF, UDM, UDR, NRF, PCF, and NSSF. Open RAN platforms implement the gNB protocol stack and interact with radio hardware or simulated radio frontends. Mixing these categories can lead to incorrect comparisons. For example, Open5GS [18], OAI-CN5G [19], and free5GC [20] are 5G CN implementations, whereas srsRAN [21] and OAI-RAN are RAN implementations.

Several open-source platforms implement the 5G Core. Open5GS implements both the 4G Evolved Packet Core (EPC) and the 5G CN. In 5G SA mode, it includes the main network functions required for standalone deployment: AMF, SMF, UPF, AUSF, UDM, UDR, NRF, PCF, and NSSF. It also provides functions such as Service Communication Proxy (SCP), Security Edge Protection Proxy (SEPP), and Binding Support Function (BSF), and uses MongoDB for the subscriber database. These features make Open5GS suitable for private 5G testbeds that require programmable SIM cards, subscriber provisioning, and integration with external gNodeB implementations [22].

OAI-CN5G is another open-source 5G Core implementation. It provides several deployment profiles, from minimal configurations with AMF, SMF, UPF, and NRF to more complete configurations with UDM, AUSF, UDR, NSSF, and PCF. OAI also provides an RAN implementation, enabling end-to-end experimentation within a single software ecosystem. Its complete deployment requires complex system configuration, especially when real-time radio processing is used [23].

free5GC is a cloud-native 5G Core implementation based on the 3GPP service-based architecture. It supports the main 5GC functions, including AMF, SMF, UPF, AUSF, UDM, UDR, NRF, PCF, and NSSF. free5CG is mostly used for research on network slicing, service-based interfaces, and cloud-native deployment models [24]. Magma [25] follows a different architecture. Instead of exposing all 5GC functions as conventional standalone Service-Based Architecture (SBA) components, it provides an open-packet core platform for scalable, distributed, and low-cost wireless access networks. This makes Magma useful for comparison in rural, edge, or enterprise scenarios, but less direct for a conventional 5G SA laboratory setup based on explicit 3GPP network functions [26].

**Figure 2:**
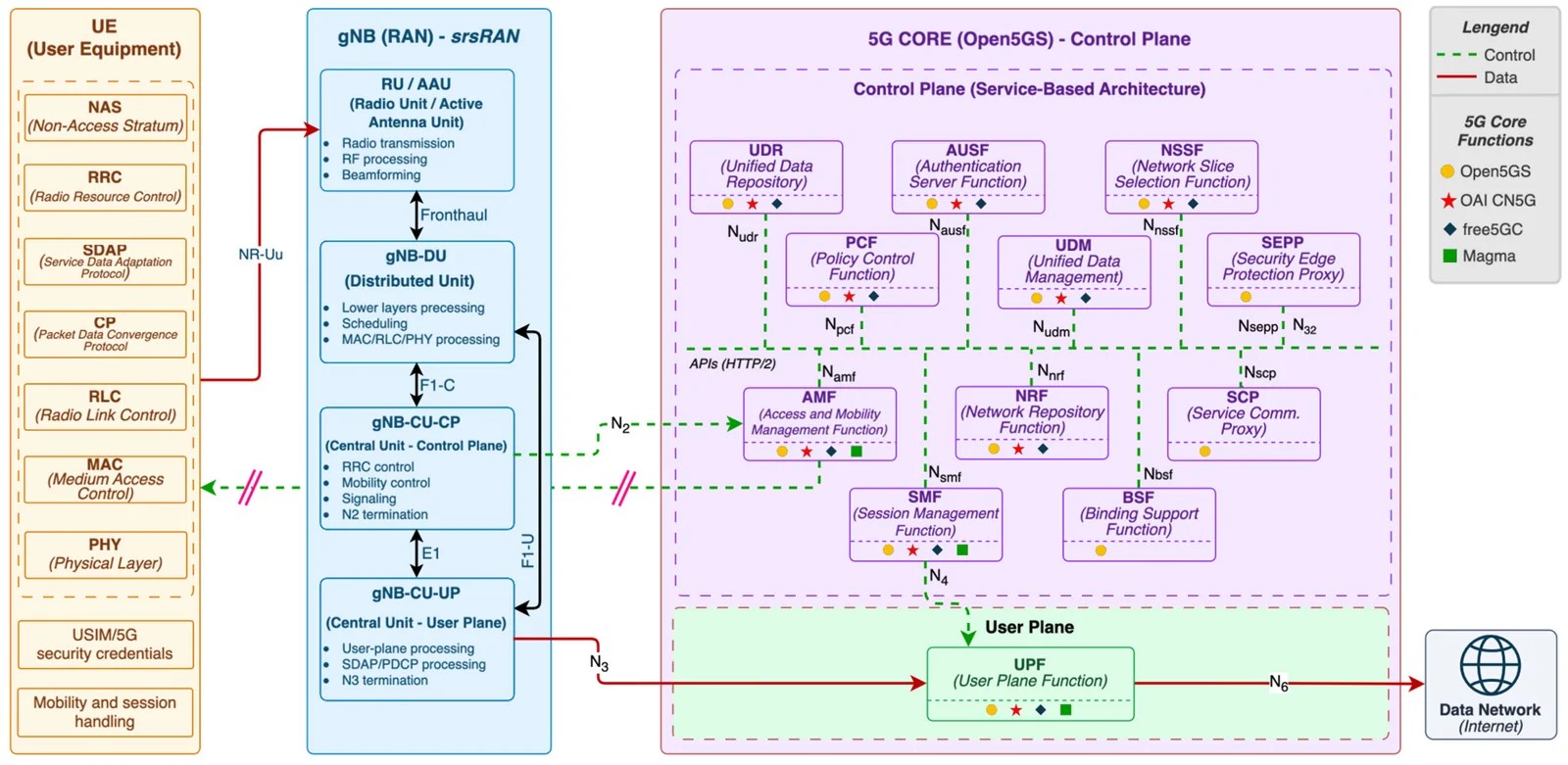
Private 5G SA architecture showing the UE, gNodeB, and 5G Core together with the main network functions and their availability across Open5GS, OAI CN5G, free5GC, and Magma.

Figure 2 shows the architecture of an OpenRAN 5G SA network and the network functions implemented by open-source 5G CN projects. The colored symbols inside each network function indicate whether Open5GS, OAI CN5G, free5GC, or Magma provides that function. The comparison shows that Open5GS covers the required 5G CN functions for the proposed experimental platform.

On the RAN side, the currently available implementations are srsRAN and OAI-RAN, which provide an open-source 5G CU/DU software stack and support integration with SDR hardware. Both can operate as gNodeBs and connect to an external 5G CN via the standard N2 and N3 interfaces, or even as an emulated UE using SDR devices without requiring a physical SIM card.

According to [27], srsRAN is a lightweight RAN implementation that meets system requirements and provides the same functionality as OAI-RAN. Accordingly, the experimental platform presented in this paper uses Open5GS for the 5G CN and srsRAN for the gNodeB, along with COTS UEs. Open5GS is selected for its support of required 5G SA core functions, subscriber management, and compatibility with external RAN implementations. srsRAN is selected for its configurable, lightweight software gNodeB, which can connect to Open5GS via N2 and N3 and to SDR hardware via UHD (USRP Hardware Driver). The testbed consists of a commercial UE, a srsRAN-based gNodeB, an Open5GS-based 5G Core, and an external data network for validation and performance measurements.

## 3. Related Works

As presented in the introduction, due to radio-spectrum regulations, most of the experimental 5G platforms often rely on RF waveguides such as splitters, multiplexers, attenuators, and coaxial cables. These setups are repeatable and avoid interference with telecom operators in licensed frequency bands, but they do not reproduce OTA radio signal propagation effects. The literature presents several implementations of OpenRAN 5G networks, the purpose of the deployments varying from security, healthcare applications, and network performance metrics.

Oliveira et al. [28] present OpenCare5G, a private 5G/Open RAN deployment for digital health applications. The paper treats private 5G networks as infrastructure for remote medical services, especially ultrasound examinations and medical data transfer between hospitals and remote sites. Its main contribution is proof-of-concept healthcare deployment based on OpenRAN principles. The paper also discusses network disaggregation, 5G CPE connectivity, and low-latency data transmission in medical scenarios.

Ahmad et al. [29] analyzed vulnerabilities in 5G Core and Open RAN components using OAI-based private network platforms. The study focuses on security evaluation rather than overall network performance. It considers two attack classes: jamming, which interferes with RF communication, and fuzzing, which injects malformed or unexpected protocol data to expose implementation weaknesses. The authors used dedicated testbeds and evaluated the impact of these threats through security evaluation procedures. The work shows that open-source 5G/Open RAN systems can be used for vulnerability analysis at the interfaces between the RAN and the CN. Its testbed includes OAI software, SDR hardware, and UE-related components, which makes it close to practical private 5G experimentation.

Stoian et al. [30] compared open-source and commercial 5G Standalone deployments under laboratory conditions. The open testbed uses Open5GS as the core network, srsRAN as the RAN software, and a USRP B210 as the SDR radio unit. The commercial-oriented setup uses the same Open5GS core, a Benetel RAN550 radio unit, and Accelleran virtualized baseband software. Throughput and latency are measured with a Motorola Edge 50 Pro terminal, and the results are compared with representative values from the literature.

Håkegård et al. [31] evaluated a 5G SA platform built with Open5GS and srsRAN on a general-purpose workstation. The study examines how radio parameters affect throughput, coverage, and latency. The authors compare measured results with theoretical expectations and 3GPP reference values. Some results are close to expected behavior, while others are limited by the SDR platform and the absence of hardware acceleration. The study also reports practical limits in UL performance, latency, and host-side processing. This work uses a similar open-source core/RAN combination to the present study, but it does not focus on isolated OTA chamber measurements or on a systematic comparison of multiple antenna configurations.

Bozis et al. [32] developed a standalone 5G platform based on OAI, commodity hardware, and USRP N310 SDR devices. The platform uses Linux scripts for startup, network configuration, connectivity checks, and measurements, which reduces the effort required to run OAI experiments. It supports both RF-simulator operation and wireless SDR-based operation, so test configurations can be evaluated before using real radio transmission. The study also reports throughput and latency under wider bandwidth configurations. This work is mainly concerned with OAI automation and general platform control. In contrast, the present paper uses Open5GS and srsRAN, validates commercial CPE and SIM operation, and studies controlled OTA performance inside a semi-anechoic chamber.

Novanana et al. [33] studied standalone non-public networks using SDR-based 4G LTE and 5G systems. The experimental setup uses Open5GS, srsRAN, USRP hardware, and a COTS UE. The authors measure throughput and latency with iPerf3 in indoor line-of-sight and non-line-of-sight conditions. Their results show higher DL and UL throughput and lower latency for the 5G SNPN (standalone non-public network) than for the LTE setup. The study also introduces a Mamdani fuzzy-logic Network Quality Index that combines SINR (Signal to Interference-plus-Noise Ratio), throughput, latency, and jitter into quality levels.

Takeda et al. [34] proposed an open-source 5G full-stack evaluation platform for testing spectral-efficiency improvements with simplified universal time-domain windowed OFDM (Orthogonal Frequency-Division Multiplexing). The platform combines SDR hardware with open-source 5G software and supports standards-based experimentation beyond link-level simulation. The study includes SDR operation and validation with a COTS UE. It also shows how a 5G implementation can be modified at the waveform and physical-layer level while keeping end-to-end connectivity.

Mukute et al. [35] benchmarked the control-plane performance and feature support of OAI-CN, Open5GS, and free5GC. The study compares popular open-source 5G CN implementations and examines their behavior during 5G procedures such as user registration. The authors connect NF operations with control-plane procedures and analyze resource use and system-call behavior. Their results show differences in supported features, processing efficiency, and implementation behavior. These results help with 5G Core selection for a private laboratory network. The study is limited to the CN side and does not test real RF transmission, SDR behavior, antenna configuration, or OTA throughput.

O’Brien et al. [36] benchmarked two private 5G SA packet-core implementations in a smart manufacturing environment. The evaluated cores are Nokia and Raemis rather than open-source platforms. The study defines practical test scenarios, including subscriber-to-subscriber communication, subscriber-to-data-center communication, UL and DL throughput, latency, high device density, and concurrent traffic. The results show that both cores can meet baseline private-network requirements, but with different behavior. One provides higher throughput, while the other is more stable under dense traffic. The study also shows how dual public land mobile network (PLMN) configurations can separate traffic according to application needs. A summarization of the reviewed papers is presented in Table 1.

The reviewed studies cover healthcare services [28], security testing [29], open and commercial deployment comparison [30], Open5GS/srsRAN platform evaluation [31], OAI automation [32], standalone non-public network quality evaluation [33], waveform-level testing [34], 5G Core benchmarking [35], and private 5G packet-core benchmarking [36]. From a technical perspective, these works differ in the adopted CNs and RAN implementations, the use of conducted or OTA radio links, support for COTS UE, antenna/MIMO configurations, and the reported performance metrics. While throughput and latency are commonly considered, radio-level indicators such as BLER and MCS are less consistently correlated with application-level measurements. Reproducibility is also addressed differently across the reviewed platforms, ranging from automated or conducted laboratory setups to wireless measurements under less controlled propagation conditions. However, most of them examine only part of the system, such as the 5G CN, security behavior, healthcare use cases, waveform design, or packet-core performance. Performance studies also tend to use a limited set of radio scenarios. They usually do not combine antenna configuration, commercial CPE/SIM validation, and isolated OTA testing in the same setup. As a result, the literature does not yet provide a private 5G SA platform evaluated inside a semi-anechoic chamber with systematic analysis of throughput, reconfigurability, and host-side limits.

The present work addresses this gap through a controlled laboratory study of a private open-source 5G SA testbed. Unlike studies based only on conducted RF paths, splitters, multiplexers, attenuators, and cabling, the semi-anechoic chamber supports controlled OTA point-to-point testing while reducing interference risk outside the test environment. This environment enables repeatable measurements while allowing more realistic UE behavior. The contribution is not only the deployment of a private network. It also shows that an open-source/private 5G architecture can be configured, attached to real SIM-based UE/CPE devices, connected to external data networks, and evaluated across controlled radio scenarios, including bandwidth and numerology changes for B5G network evaluation.

## 4. Experimental Setup

**Figure 3:**
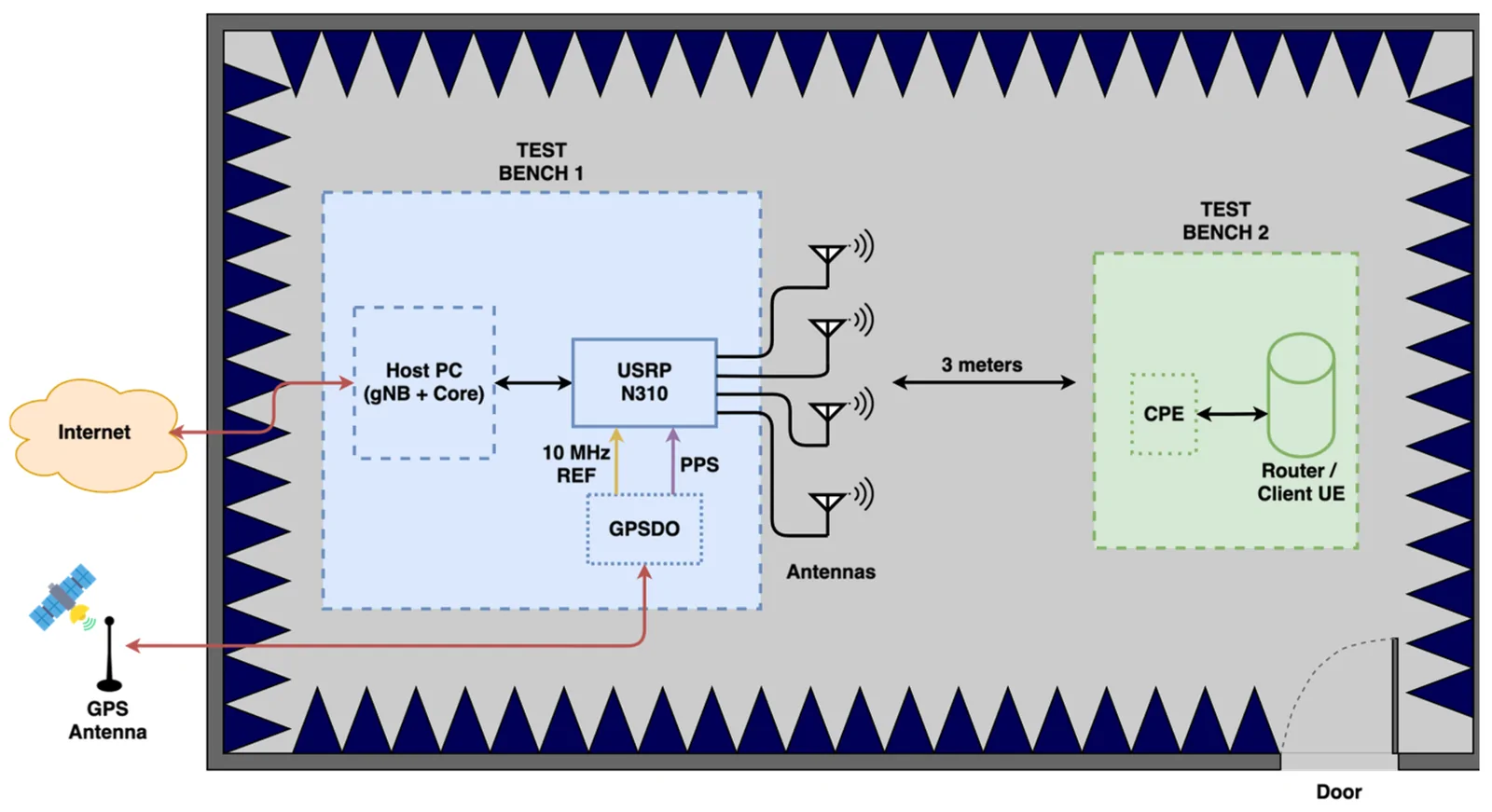
Architecture of the implemented open-source RAN 5G SA network.

The experimental architecture is presented in Figure 3. As can be seen, the whole setup is deployed in a TDK 3M semi-anechoic chamber of the EMC lab [37] at the Stefan cel Mare University of Suceava. The 5G CN and the gNB are deployed on the same host machine, while the radio frontend is deployed on an Ettus USRP N310 SDR. This design minimizes internal latency between the RAN and core on the N2 and N3 interfaces and simplifies the integration of Open5GS and srsRAN. The N2 interface carries control-plane signaling toward the AMF, while N3 carries GTP-U user-plane traffic toward the UPF. A commercial 5G CPE Nokia Fastmile 5G receiver acts as the UE, and a programmable SIM is configured to match the test PLMN and subscriber database. The distance between the UE and the gNB is approximately 3 m. The endpoint used for measurements is a PC connected through a Nokia Wi-Fi Beacon downstream of the 5G CPE.

The hardware configuration of the host machine level consists of a workstation with an Intel i9-14900 CPU with 24 cores, 32 GB of RAM, and two network interface cards. An ASUS XG-C100F card with a 10 Gbit SFP+ port connects the core network host to the external Internet. A Mellanox MCX631102AS-ADAT card with two 10 Gbit SFP+ ports connects the SDR radio interface to the radio frontend. The radio frontend, represented by an Ettus USRP N310, is an SDR platform with four transmit and four receive channels, which enables SISO (Single Input, Single Output), 2 × 2 MIMO (Multiple-Input and Multiple-Output), and up to 4 × 4 MIMO configurations. A GPSDO device generates the 10 MHz REF clock and the PPS signal required to reduce the carrier frequency error and frequency drift for connection with COTS UE devices.

The UE side consists of a Nokia FastMile 5G CPE and a sysmoISIM-SJA5 programmable SIM card. The CPE provides 5G radio access, while the Nokia Wi-Fi Beacon creates a local LAN for the PC on which measurements are performed. This arrangement allows the test endpoint to generate application-layer traffic while the 5G CPE translates the local traffic into the radio link toward the gNodeB. The Wi-Fi link was used only as the local access segment between the measurement PC and CPE and was not independently characterized; therefore, it’s possible contribution to the end-to-end traffic generation cannot be separated from the overall measurement path.

Open5GS provides the 5G CN and is configured through its NFs and subscriber database. The implementation includes the AMF, SMF, UPF, UDM/UDR, AUSF, and NRFs required by the 5G SA architecture. MongoDB and the Open5GS WebUI are used for subscriber management. The SIM card parameters are programmed with pySim [38] and then inserted into the core database through the International Mobile Subscriber Identity (IMSI), OPC, and related authentication data. srsRAN implements gNodeB and communicates with Open5GS through the localhost AMF address configured in the gNB. Its configuration includes the PLMN, tracking area, ARFCN, radio bandwidth, sampling rate, and antenna settings. For reproducibility of the open-source 5G SA testbed, Table 2 provides an overview of the software and firmware versions used in the experimental platform, as well as the relevant host system and device configuration parameters.

**Figure 4:**
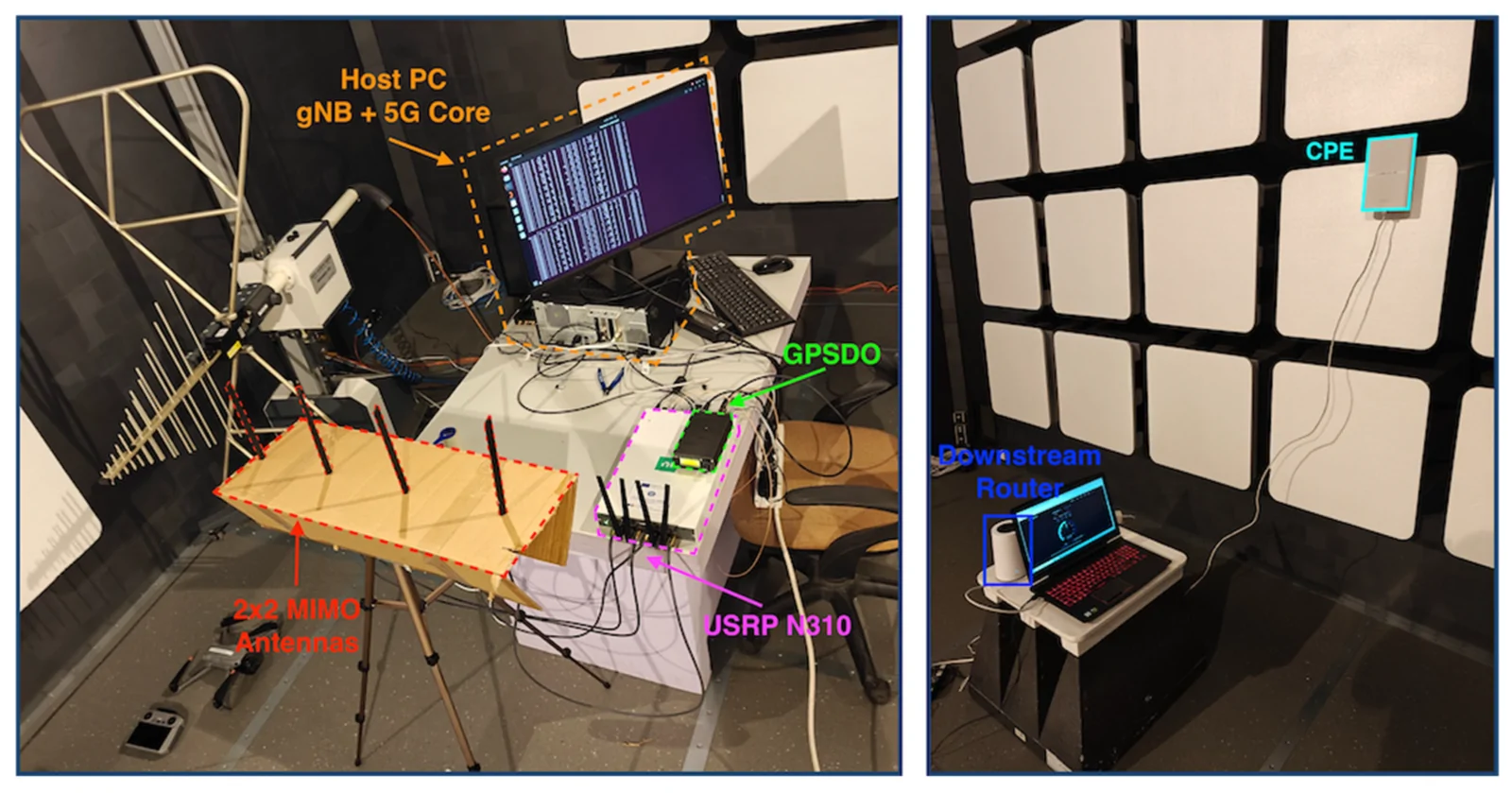
Practical implementation of the 5G testbed setup in the semi-anechoic chamber.

Figure 4 presents the semi-anechoic chamber setup. The role of the semi-anechoic chamber is to enable OTA point-to-point measurements while avoiding interference with other telecom operators and evaluating the performance of the network in OTA mode. In a standard laboratory deployment, radio waves are affected by reflections from walls and surrounding objects, uncontrolled attenuation, and signals generated by the networks of other telecom operators. An anechoic chamber removes these variables by using radiation-absorbing structures, typically carbon-loaded pyramidal materials, and by enclosing the chamber in a conductive shell that behaves like a Faraday cage. This produces a stable radio environment and makes the measurements repeatable.

This setup is particularly useful for distinguishing between channel-related limitations and platform-related limitations. If the propagation conditions are controlled, the evaluations can determine whether observed throughput limits are caused by the server, the SDR interface, the gNodeB scheduler, or the UE. The chamber also enables more precise evaluation of MIMO by changing antenna positions or by adding a programmable channel emulator.

The network was configured with the test PLMN MCC 001 and MNC 01. The same values were provisioned in the Open5GS subscriber database, srsRAN configuration, and programmable SIM to ensure consistent identification and authentication within the private test network. UE connectivity is provided through the TUN and ogstun interface used by Open5GS. To enable traffic routing, IP redirection mechanisms are enabled for both IPv4 and IPv6, and network address translation (NAT) rules are configured using the iptables utility. Thus, packets originating from the address pool allocated to UE devices can be translated and routed to external networks.

The radio frontend is configured in the FR1 band, as the USRP N310 SDR supports operation at frequencies up to 6 GHz. The evaluation methodology combines validation of the UE connection to the network, monitoring of the radio link, generation of data traffic and analysis of the obtained results.

Before performing the performance measurements, the log messages generated by the gNB are monitored to confirm the establishment of the radio connection, the allocation of the RNTI (Radio Network Temporary Identifier) and the creation of the PDU session context. The measurements are performed exclusively after the UE has been successfully attached to the network.

**Figure 5:**
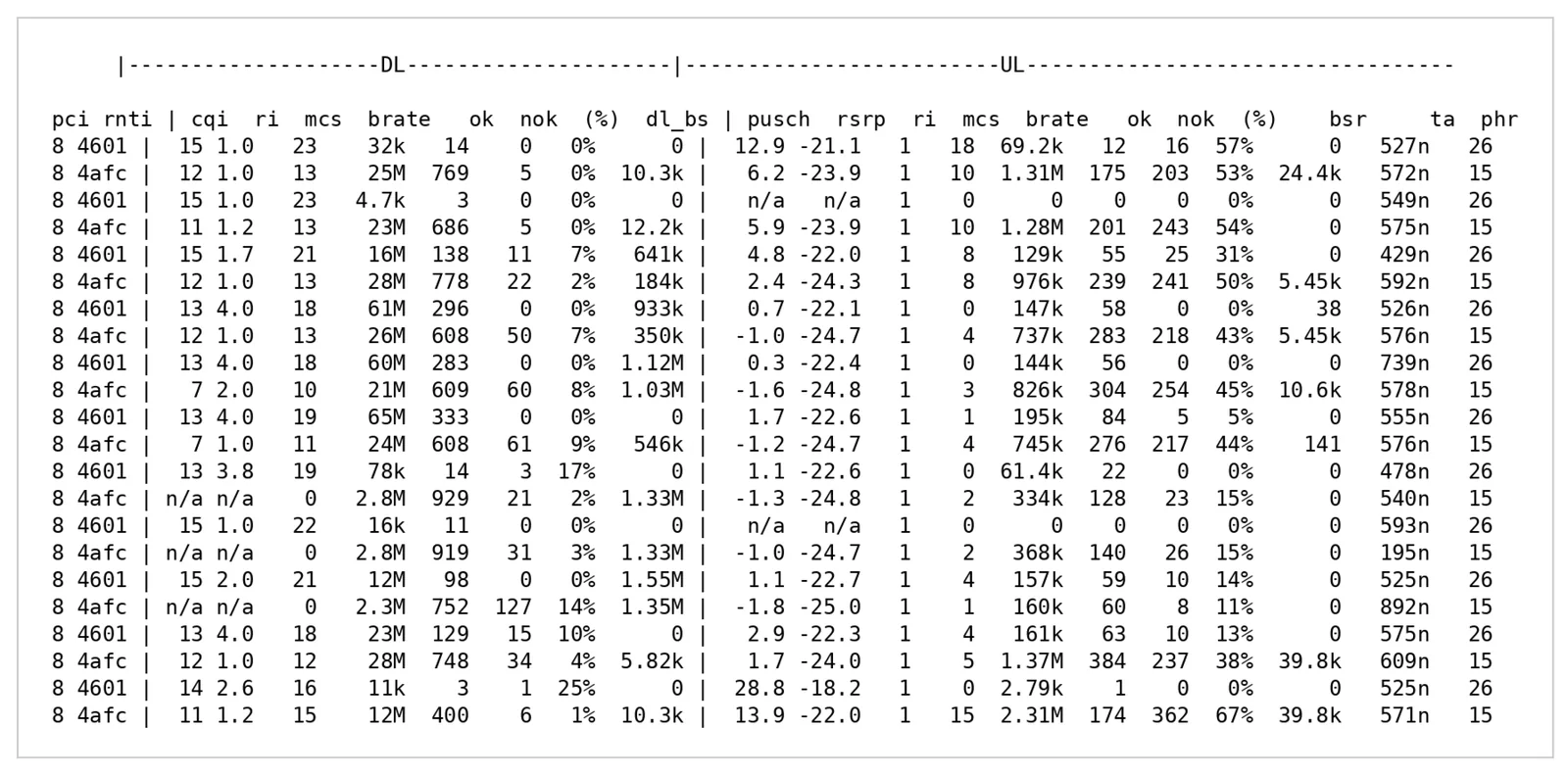
gNB trace logs provided by the srsRAN.

The performance indicators were extracted from the gNB trace logs for each UE. As shown in Figure 5, for each RNTI, the gNB systematically reports the downlink (DL) and uplink (UL) metrics, namely the channel quality indicator (CQI), rank indicator (RI), modulation and coding scheme (MCS), bitrate, number of successfully decoded packets (OK), number of failed packets (NOK), block error rate (BLER), and downlink buffer status (DL BS). For the UL, the reported indicators include the PUSCH (Physical Uplink Shared Channel) signal quality, RSRP (Reference Signal Received Power), RI, MCS, bitrate, number of OK and NOK packets, BLER, buffer status report (BSR), timing advance (TA), and power headroom report (PHR). For this implementation, the main performance indicators taken into consideration are DL throughput, UL throughput, BLER, and MCS distribution.

Performance metrics are extracted for multiple communication scenarios. We first evaluated the TDD and FDD configurations. Secondly, for each duplex configuration, we evaluated multiple MIMO configurations, including SISO, 2 × 2 MIMO, and 4 × 4 MIMO. For each evaluated scenario, DL and UL user-plane traffic were generated from the measurement PC using the fast.com web-based speed-test service. The generated traffic traversed the end-to-end communication path from the measurement PC through the Nokia Wi-Fi Beacon, Nokia FastMile 5G CPE, OTA radio link, srsRAN gNB, Open5GS 5G Core, and external data network.

The FDD duplex is configured in the n7 band with an ARFCN of 534000 for DL, representing a central frequency of 2670 MHz, a duplex spacing of 120 MHz, and the UL at the central frequency of 2550 MHz. The TDD is configured in the n78 band with an ARFCN of 646666, with a central frequency of 3.7 GHz. The antennas used in this setup are general-purpose omnidirectional antennas, which are spatially separated with a minimum of λ/2 in order to decrease the mutual coupling between adjacent channels in the MIMO mode [39].

For each evaluated configuration, one measurement session was performed. The average throughput reported in the following section represents the temporal average of the gNB-reported bitrate over the corresponding measurement interval, while the maximum throughput represents the highest instantaneous value observed during that interval.

## 5. Results and Discussion

This section compares the performance of the implemented 5G SA testbed for FDD and TDD operation using 1 × 1, 2 × 2, and 4 × 4 antenna configurations. The analysis considers the average throughput, maximum throughput, and BLER measured separately for the DL and UL. Additionally, these are correlated with the MCS distributions for each of the considered configurations.

**Figure 6:**
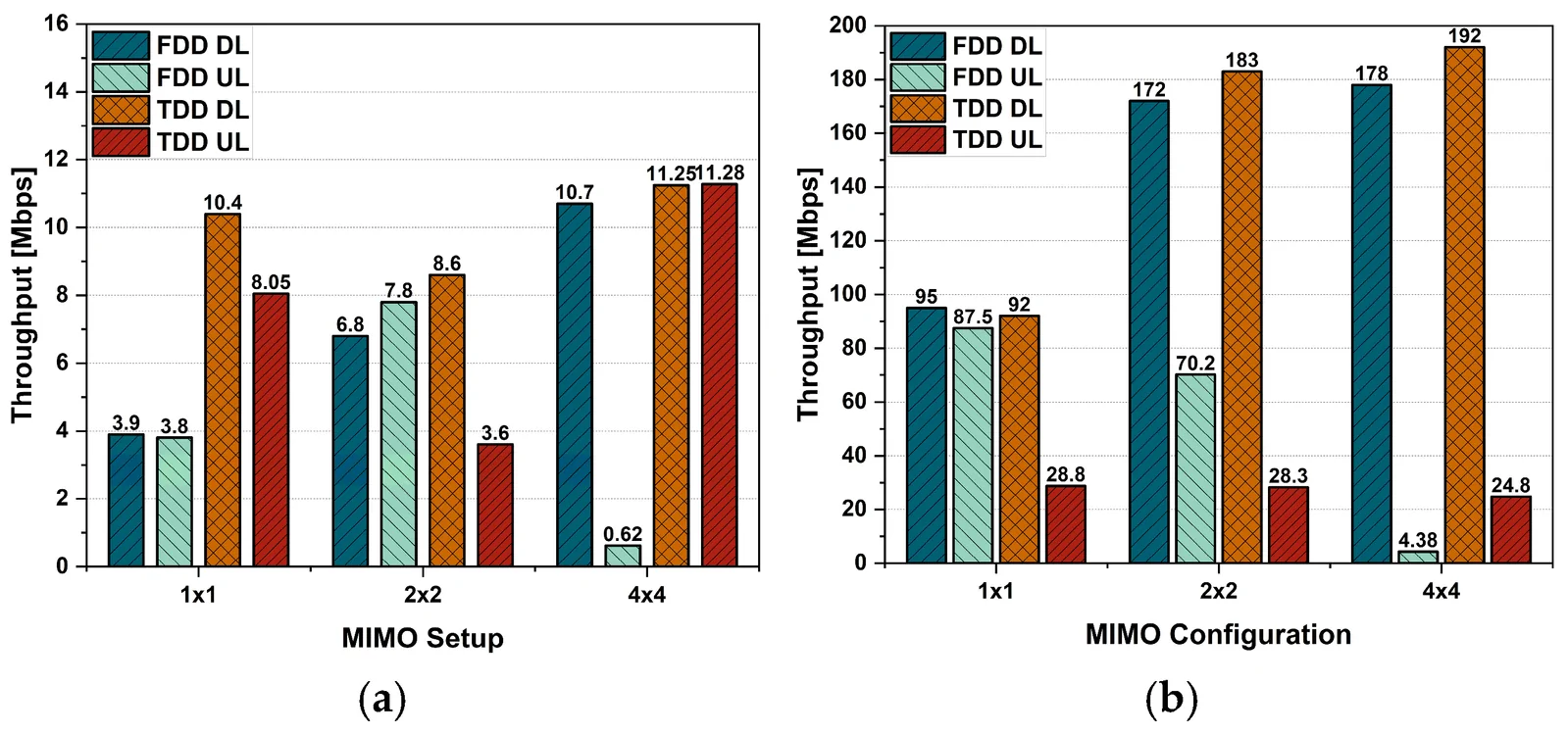
5G SA network performance metrics: (**a**) average throughput; (**b**) maximum throughput.

In Figure 6a, we can see the average throughput, representing the sustained data rate. For the FDD DL, the average throughput increases progressively from approximately 3.9 Mbps for the 1 × 1 configuration to 6.8 Mbps for 2 × 2 and 10.7 Mbps for 4 × 4. As can be seen, DL performance in the FDD mode increases as the number of antennas in the MIMO configuration increases. The improvement from 1 × 1 to 2 × 2 is approximately 74%, while the 4 × 4 configuration provides an additional 57% increase in throughput compared to 2 × 2. The TDD DL average throughput values do not follow the same trend. As seen in Figure 6a, the average throughput values are 10.4 Mbps for 1 × 1, 8.6 Mbps for 2 × 2, and 11.25 Mbps for 4 × 4, suggesting that the number of configured antennas is not the only factor determining a 5G network’s throughput. For the UL, the FDD results show an increase from approximately 3.8 Mbps in the 1 × 1 MIMO to 7.8 Mbps in the 2 × 2 MIMO configuration. However, the average throughput decreases to approximately 0.62 Mbps for the 4 × 4 configuration. This decrease in average throughput shows that using four radio paths does not result in an increase in the number of usable UL spatial layers. The TDD UL average throughput also varies between the evaluated configurations. The obtained values are approximately 8.05 Mbps for 1 × 1, 3.6 Mbps for 2 × 2, and 11.28 Mbps for 4 × 4. The 4 × 4 configuration provides the highest measured TDD UL average throughput, while the 2 × 2 case presents the lowest value. In addition, because TDD UL and DL transmissions share the same carrier in time, the available UL throughput depends on the TDD slot configuration.

Figure 6b shows the maximum throughput for each evaluated configuration. Compared to the average throughput metric, the maximum throughput represents a short-duration peak of the highest instantaneous data rate supported by the 5G network rather than the continuously sustainable rate. The maximum FDD DL throughput increases from approximately 95 Mbps for 1 × 1 to 172 Mbps for 2 × 2 and 178 Mbps for 4 × 4. The maximum TDD DL throughput increases from approximately 92 Mbps for 1 × 1 to 183 Mbps for 2 × 2 and 192 Mbps for 4 × 4. The results show that DL throughput improves when scaling from SISO to 2 × 2 MIMO. However, the improvement from 2 × 2 to 4 × 4 is relatively limited, at approximately 3.5% for FDD and 4.9% for TDD. This limited throughput gain suggests that the four configured RF paths do not continuously produce four independent spatial streams. The controlled line-of-sight environment inside the semi-anechoic chamber may also produce correlated propagation paths. Although this environment improves repeatability, it does not provide the multipath conditions required to maximize the capacity gain of 4 × 4 MIMO, meaning that a nominal 4 × 4 MIMO configuration may behave more like 2 × 2 MIMO, providing diversity gain but little additional spatial multiplexing gain.

For the UL, the maximum FDD throughput decreases from approximately 87.5 Mbps for 1 × 1 to 70.2 Mbps for 2 × 2 and approximately 4.4 Mbps for 4 × 4. The 4 × 4 FDD UL throughput value is consistent with the reduction in average throughput. The maximum TDD UL throughput is more stable, with values of approximately 28.8, 28.3, and 24.8 Mbps for the 1 × 1, 2 × 2, and 4 × 4 configurations, respectively.

As can be seen, for the DL scenarios there is a large difference between the maximum and average throughput. Although peak values of 180–190 Mbps are obtained, the average throughput remains considerably lower. This indicates that the high-rate transmission conditions are maintained only for limited periods. Variations in PRB allocation, selected MCS, transmission rank, retransmissions, traffic generation, and scheduler operation can reduce the sustained throughput relative to the measured maximum.

**Figure 7:**
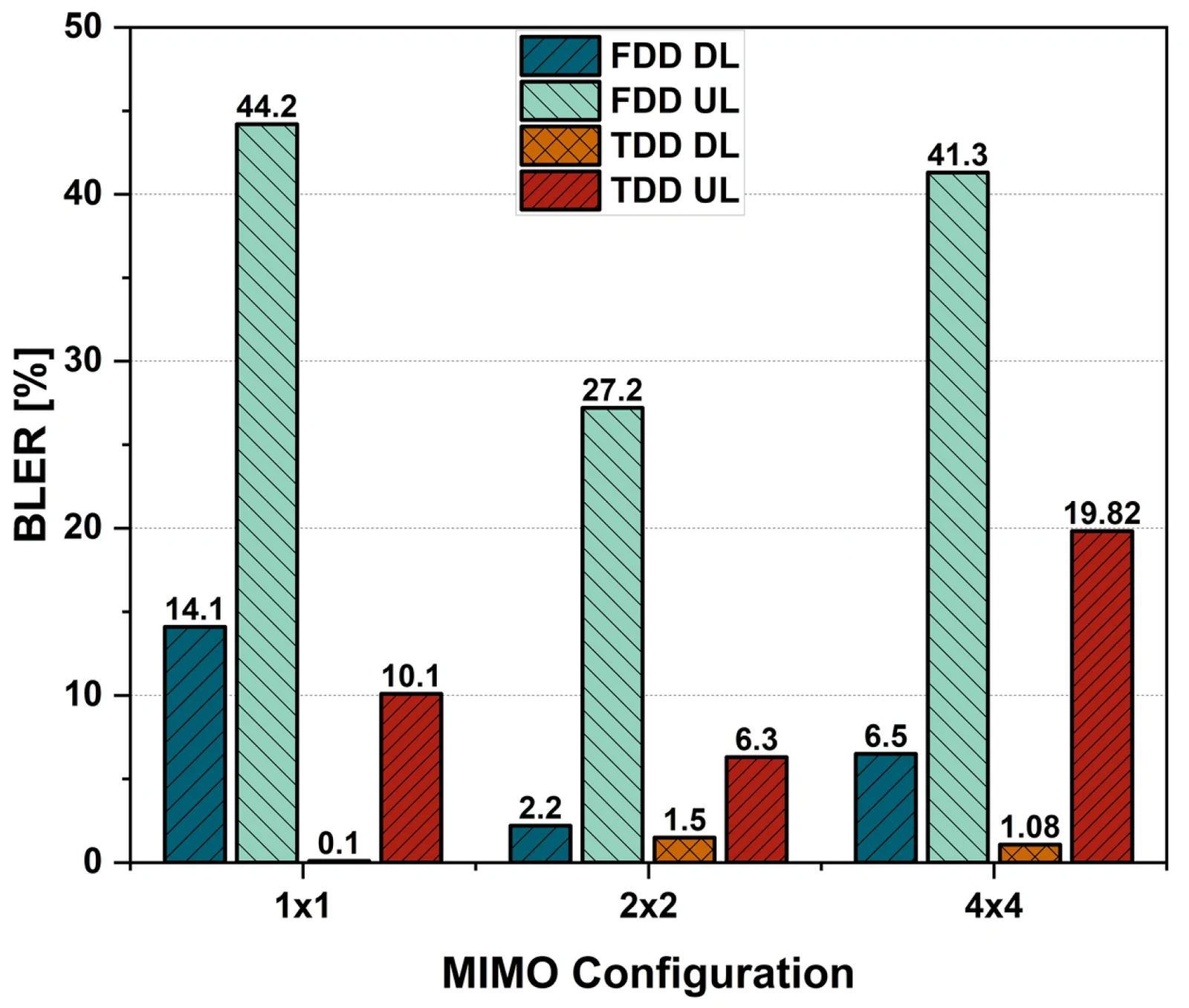
5G SA network performance metrics: BLER.

The next evaluated metric is the BLER, the values for which are presented in Figure 7. For the FDD DL, BLER decreases from approximately 14.1% for 1 × 1 to 2.2% for 2 × 2 and reaches 6.5% for 4 × 4. The TDD DL has the lowest BLER values: approximately 0.1% for 1 × 1, 1.5% for 2 × 2, and 1.08% for 4 × 4, suggesting limited HARQ retransmission overhead and a stable DL radio connection. The UL BLER is higher than the DL BLER. For FDD, the BLER values are approximately 44.2% for 1 × 1, 27.2% for 2 × 2, and 41.3% for 4 × 4. The FDD 2 × 2 configuration has the highest average UL throughput among FDD cases, although its BLER is relatively high. For TDD UL, BLER values of approximately 10.1%, 6.3%, and 19.8% are obtained for 1 × 1, 2 × 2, and 4 × 4, respectively. The 2 × 2 configuration provides the lowest TDD UL BLER, whereas the 4 × 4 configuration provides the highest average throughput but also the highest BLER.

**Figure 8:**
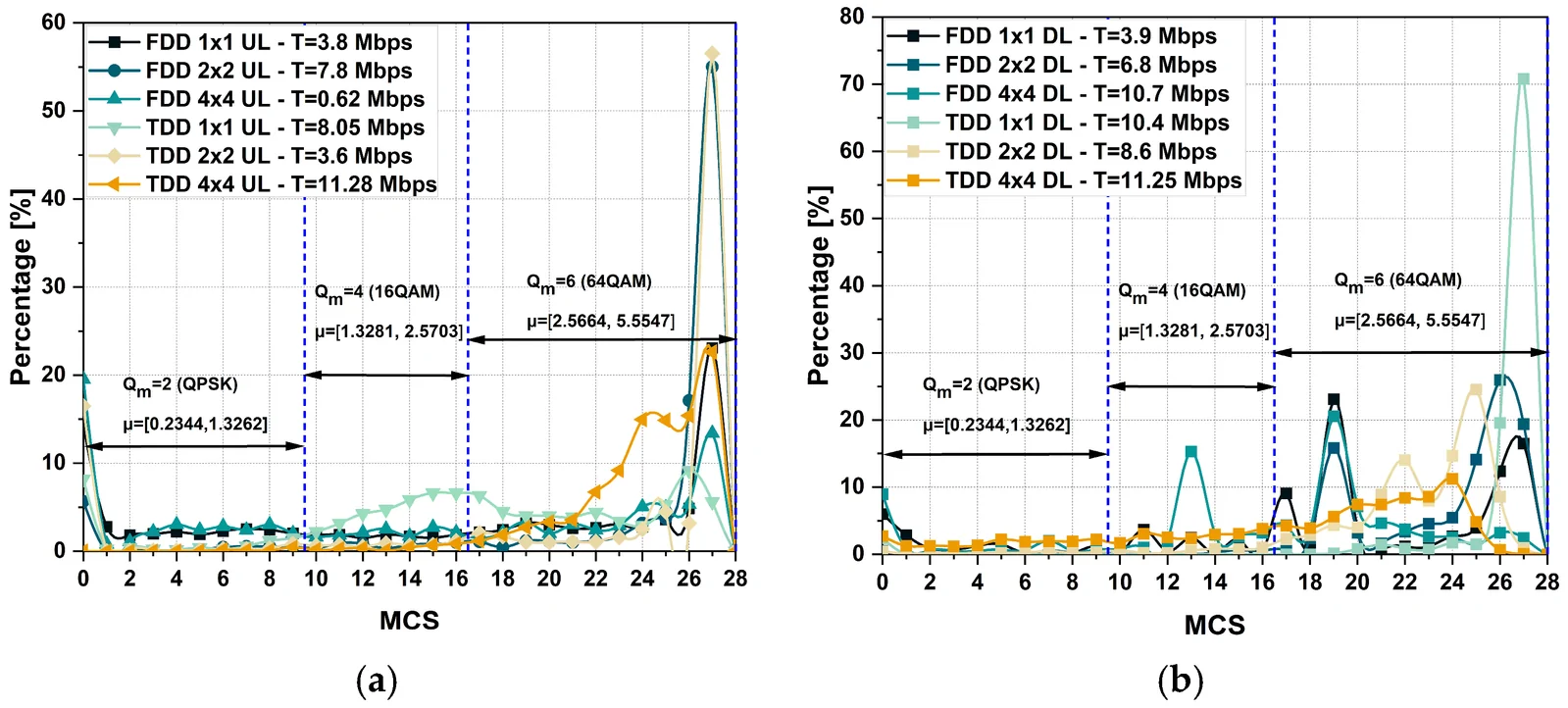
Distribution of the MCS values for (**a**) uplink and (**b**) downlink.

The last evaluated metric is presented in Figure 8, which shows the distribution of the MCS values for UL and DL 5G traffic. The network was configured to use the 64QAM table for MCS selection, meaning that the MCS values ranging from 0 to 9 represent the use of QPSK modulation and spectral efficiency µ ranging from 0.2344 to 1.3262, MCS values ranging from 10 to 16 use 16QAM modulation and a *µ* ranging from 1.3281 to 2.5703, and finally MCS values starting from 17 to 28 use 64QAM modulation and have a µ ranging from 2.5664 to 5.5547. The gNB varies the MCS based on measurements of the radio communication channel and the results of previous radio transmissions. For DL, the MCS selection is primarily based on the channel-state information (CSI) reported by the UE, whereas for UL, the gNB estimates the channel quality from PUSCH, DM-RS, or SRS measurements.

Figure 8a presents the UL MCS distributions. As can be seen, the UL is distributed across a relatively wide range of MCS indices, specifically the FDD configurations, indicating that the scheduler continuously adapts the MCS according to the estimated UL channel conditions. Some configurations, such as 1 × 1, 2 × 2 and 4 × 4 TDD, are concentrated in the high-MCS region, meaning higher-order modulations are used. The UL results also show that using a high MCS does not necessarily guarantee a high average throughput. This can be seen in the case of the FDD 4 × 4 configuration, where even if 20% of all the MCSs used are above 17 (64 QAM), the average throughput is only 0.62 Mbps due to the high BLER. Consequently, the MCS distribution must be interpreted together with BLER and throughput.

Figure 8b shows the DL MCS distributions. As can be seen, for DL, the gnB uses higher MCS values compared to UL. Several DL configurations, such as 1 × 1 FDD, 4 × 4 FDD, 1 × 1 TDD, and 4 × 4 TDD, have peaks in the high-MCS region. The TDD 1 × 1 and 4 × 4 DL use high MCS values and obtain BLER values close to 1% or lower. The wider MCS distributions observed in some configurations indicate frequent link adaptation, potentially caused by changes in resource allocation, effective rank, or instantaneous channel conditions.

Overall, the results show that 2 × 2 MIMO provides the most consistent performance improvement, especially for FDD operation, as it significantly increases the maximum DL throughput and obtains the lowest FDD BLER values. The 4 × 4 configuration provides the highest average and maximum DL throughput, but the increase in throughput over 2 × 2 is relatively small.

The results also show that a 5G network testbed cannot be evaluated only by average throughput and maximum throughput, as additional network metrics such as MCS and BLER provide additional information on the network behavior. Maximum throughput describes the short-term capability of the radio interface, whereas average throughput shows the sustained performance. MCS indicates the spectral efficiency selected by the scheduler, while BLER reveals whether the selected transmission parameters can be reliably maintained. As can be seen from the presented results, a configuration that reaches a high MCS or a high instantaneous bitrate may still obtain a limited average throughput when HARQ, restricted resource allocation, or rank limitations are imposed.

Under the evaluated conditions, 2 × 2 MIMO provided the most balanced operating point for FDD. The 4 × 4 configuration reaches the highest DL throughput values, but it has a limited gain over the 2 × 2 configuration. These findings also support the broader objective of the work: validating an end-to-end, open-source 5G SA platform that integrates Open5GS, srsRAN, an SDR-based radio frontend, and commercial CPE equipment in a controlled OTA environment. The testbed successfully supports UE registration, PDU-session establishment, external data connectivity, radio-parameter reconfiguration, and the extraction of detailed gNB-level performance indicators, thereby addressing the identified need for repeatable OTA evaluation using COTS equipment rather than conducted RF connections alone.

At the same time, the results define the practical limits of the present implementation. The significant separation between maximum and average throughput shows that the platform can briefly support high data rates, but these conditions are not maintained throughout the complete measurement interval. Moreover, because the FDD and TDD experiments are performed in different NR bands and use different duplex-resource structures, their results should be interpreted as an evaluation of the complete configurations rather than as an isolated comparison of the duplexing mechanisms. Overall, the study confirms that the developed platform is suitable for controlled 5G SA experimentation and shows that effective MIMO rank, link adaptation, retransmission behavior, duplex configuration, and UE limitations must be considered when evaluating the performance of open-source SDR-based 5G networks.

## 6. Conclusions

This paper presented an open-source private 5G Standalone testbed designed for controlled over-the-air experimentation, application validation, and network development. The platform integrates an open-source 5G Core and RAN with software-defined radio hardware, programmable subscriber credentials, and commercial user equipment. It provides access to the complete communication chain, including UE registration and authentication, PDU-session establishment, user-plane routing, radio transmission, and external data connectivity.

Although the implemented system is based on 5G SA technologies, its purpose extends to a configurable and observable testbed for developing and evaluating applications, network functions, and control mechanisms for beyond-5G systems. The combination of throughput measurements, BLER, MCS, packet-decoding statistics, and gNB traces enables cross-layer analysis of the relationship between application performance and radio-network behavior. Performance degradation can therefore be investigated rather than just observed. The collected measurements help determine whether service-level variations are associated with radio-link instability, retransmissions, scheduling behavior, resource allocation, UE characteristics, or limitations of the software and host platform. This diagnostic capability can be used for both the optimization of current private 5G deployments and the development of future B5G network-control mechanisms. In the conducted tests, the FDD average DL throughput increased by approximately 74% from 1 × 1 to 2 × 2 and by a further 57% from 2 × 2 to 4 × 4. However, the maximum DL throughput increased by only approximately 3.5% for FDD and 4.9% for TDD when moving from 2 × 2 to 4 × 4, confirming the limited additional gain of the 4 × 4 configuration under the evaluated conditions.

The semi-anechoic chamber provides stable and repeatable propagation conditions, allowing experiments to be repeated after software, hardware, or configuration changes without uncontrolled external interference. At the same time, the OTA connection preserves the interaction between the software-defined radio frontend and commercial user equipment. The platform fills the experimental gap between simulation, conducted RF testing, and measurements performed in uncontrolled operational environments. The present evaluation also has several limitations. The measurements were performed with a static CPE under fixed point-to-point OTA conditions, and mobility effects were therefore not considered. Dedicated latency and jitter measurements were not performed because the adopted end-to-end traffic path includes the local WLAN segment and an external Internet service, which would prevent the measured delay from being attributed exclusively to the 5G SA testbed.

Overall, the developed testbed provides a reproducible framework for validating private 5G services and for studying the evolution of open-source RAN platforms toward beyond-5G networks. Its modular architecture can support future work on intelligent RAN optimization, closed-loop network automation, enhanced network slicing, and application-aware resource allocation. The testbed can therefore be used to compare configurations, identify performance limitations, validate new network mechanisms, and verify service behavior before deployment in industrial, academic, or private-network environments.

## Figures and Tables

**Table 1:** Technical comparison of related private 5G and open-source testbeds. N/A indicates that the parameter is not applicable; N/R indicates that it was not reported; and VAR indicates that the paper reports multiple scenario-dependent values without a single representative result.

Work	RAN	Core	Evaluation	Band	BW/RBs	SCS	MIMO	RTT/ Latency, ms	Throughput [DL, UL], Mbps	BLER
Oliveira [28]	Airspan O-RAN, split 7.2x	Virtualized 5GC	Digital-health PoC	n77	100 MHz	N/R	4T4R	17–48	Up to [11.95, 10.21]	N/R
Ahmad [29]	OAI/Open RAN	OAI 5GC	Jamming and fuzzing	N/R	N/R	N/R	N/R	N/A	N/A	N/R
Stoian [30]	srsRAN	Open5GS	Speed test/ping	n78	20 MHz, 51 RBs	30 kHz	N/R	47.4 mean; 28 min	[28, 14.4] mean; [41, 18] peak	N/R
Stoian [30]	Accelleran/Benetel RAN550	Open5GS	Speed test/ping	n78	100 MHz, 273 RBs	30 kHz	N/R	43.4 mean; 27 min	[296.4, 32.6] mean; [490, 49] peak	N/R
Håkegård [31]	srsRAN	Open5GS	iPerf/ping	n77	40 MHz	30 kHz	1 × 1, 2 × 2	17.3 mean; 5.3 min	[123, 39] best mean	N/R
Bozis [32]	OAI gNB	OAI 5GC	iPerf/ping	n78	20–60 MHz; 51–162 RBs	30 kHz	SISO	9.19–9.89 mean; 6.78 optimized	Cabled: [38.92–97.88, 21.10–67.01]; wireless: [24.08–89.50, 16.87–59.17]	N/R
Novanana [33]	srsRAN	Open5GS	iPerf3; LOS/NLOS	N/R	N/R	N/R	N/R	VAR	VAR	N/R
Takeda [34]	Modified OAI	OAI 5GC	COTS-UE DL test	4.849 GHz	100 MHz	30 kHz	1 × 1	N/R	[292.3, N/R] for CP-OFDM; DL decreases to 36.2 with largest UTW	N/R
Mukute [35]	UE/gNB traffic emulator	OAI, Open5GS, free5GC	UE-registration benchmark	N/A	N/A	N/A	N/A	N/A	N/A	N/A
O’Brien [36]	Commercial Private 5G RAN	Nokia/ Raemis	Industrial traffic scenarios	N/R	N/R	N/R	N/R	VAR	VAR	N/R
**This work**	srsRAN	Open5GS	Speed test	n78, n7	20 MHz	30 kHz	1 × 1, 2 × 2, 4 × 4	N/R	[178, 87.5] peak FDD [192, 28.8] peak TDD	VAR

**Table 2:** Configuration of the experimental 5G SA testbed.

Component/Parameter	Version/Configuration
Core Network	Open5GS v2.7.6-42-g59391d8d5
Radio Access Network	srsRAN release_25_10-192-g4bf1543936
USRP Hardware Driver	UHD 4.3.0
Radio Frontend	Ettus USRP N310, FPGA XG (2 × 10 Gbit SFP+)
Host operating system	Ubuntu 22.04.5 LTS Kernel 6.8.0-136-generic
CPU frequency and governor	CPU Governor: Performance CPU Frequency: 5.5 GHz
UE	Nokia FastMile 5G CPE SW: 1.2404.00.0755
Programable SIM	sysmoISIM-SJA5

## Data Availability

The original contributions presented in this study are included in the article. Further inquiries can be directed to the corresponding author.

## Abbreviations

The following abbreviations are used in this manuscript:

*5G SA*	Fifth-Generation Standalone
*AI*	Artificial Intelligence
*AMF*	Access and Mobility Management Function
*AUSF*	Authentication Server Function
*BLER*	Block Error Rate
*BSF*	Binding Support Function
*BSR*	Buffer Status Report
*CN*	Core Network
*COTS*	Commercial off-the-shelf
*CPE*	Customer-premises equipment
*CPU*	Central Processing Unit
*CQI*	Channel Quality Indicator
*DL*	Downlink
*DL BS*	Downlink buffer status
*DU*	Open Distributed Unit
*EPC*	Evolved Packet Core
*FDD*	Frequency Division Duplexing
*gNB*	gNodeB
*GPSDO*	GPS Disciplined Oscillator
*GTP*	GPRS Tunneling Protocol User Plane
*HARQ*	Hybrid Automatic Repeat reQuest
*IAB*	Integrated Access and Backhaul
*IMSI*	International Mobile Subscriber Identity
*LTE*	Long-Term Evolution
*MAC*	Medium Access Control
*MCC*	Mobile Country Code
*MCS*	Modulation and Coding Scheme
*MIMO*	Multiple-Input and Multiple-Output
*ML*	Machine Learning
*MNC*	Mobile Network Code
*NAS*	Non-Access Stratum
*NAT*	Network Address Translation
*NF*	Network Function
*NOK*	Number of Failed Packets
*NPNs*	Non-Public Networks
*NR*	New Radio
*NRF*	Network Repository Function
*NSA*	Non-Standalone
*NSSF*	Network Slice Selection Function
*NTNs*	Non-Terrestrial Networks
*O-CU*	Open Central Unit
*O-RU*	Open Radio Unit
*OAI*	Open Air Interface
*OFDM*	Orthogonal Frequency-Division Multiplexing
*ORAN*	Open Radio Access Network
*OTA*	Over-the-Air
*PCF*	Policy Control Function
*PDCP*	Packet Data Convergence Protocol
*PDU*	Protocol Data Unit
*PHR*	Power Headroom Report
*PHY*	Physical Layer
*PLMN*	Public Land Mobile Network
*PPS*	Pulses Per Second
*PUSCH*	Physical Uplink Shared Channel
*RAN*	Radio Access Network
*RF*	Radio Frequency
*RI*	Rank Indicator
*RIC*	RAN Intelligent Controller
*RLC*	Radio Link Control
*RNTI*	Radio Network Temporary Identifier
*RRC*	Radio Resource Control
*RSRP*	Reference Signal Received Power
*RTT*	Round Trip Time
*SBA*	Service-Based Architecture
*SCP*	Service Communication Proxy
*SDAP*	Service Data Adaptation Protocol
*SDR*	Software-Defined Radio
*SEPP*	Security Edge Protection Proxy
*SFP*	Small-Form-Factor Pluggable
*SIM*	Subscriber Identity Module
*SINR*	Signal to Interference-Plus-Noise Ratio
*SISO*	Single Input, Single Output
*SMF*	Session Management Function
*SNPN*	Standalone Non-Public Network
*TA*	Timing Advance
*TDD*	Time Division Duplexing
*TSN*	Time-Sensitive Communications
*TUN*	Tunneling
*UDM*	Unified Data Management
*UDR*	Unified Data Repository
*UE*	User Equipment
*UHD*	USRP Hardware Driver
*UL*	Uplink
*UPF*	User Plane Function
*URLLC*	Ultra-Reliable Low-Latency Communications
